# Ecotoxicity Evaluation of Industrial Waste and Construction Materials: Comparison Between Leachates from Granular Steel Slags and Steel Slags-Containing Concrete Through a Plant-Based Approach

**DOI:** 10.1007/s00128-023-03764-y

**Published:** 2023-06-21

**Authors:** Carlotta Alias, Ilaria Zerbini, Alessandro Abbà, Laura Benassi, Umberto Gelatti, Sabrina Sorlini, Giovanna Piovani, Donatella Feretti

**Affiliations:** 1 Department of Medical and Surgical Specialties, Radiological Sciences and Public Health, University of Brescia, viale Europa 11, 25123 Brescia, Italy; 2 Department of Civil, Environmental, Architectural, Engineering and Mathematics, University of Brescia, via Branze 43, 25123 Brescia, Italy; 3 B+LabNet—Interdepartmental Sustainability Lab, University of Brescia, via Branze 45, 25123 Brescia, Italy; 4 Department of Molecular and Translational Medicine, University of Brescia, viale Europa 11, 25123 Brescia, Italy

**Keywords:** *Allium cepa*, *Cucumis sativus*, *Lepidium sativum*, Waste reuse, Germination, Phytotoxicity

## Abstract

Steel slags, the main waste product from the steel industry, may have several reuse possibilities. Among others, building applications represent a crucial field. However, the potential impact of harmful substances on the environment should be assessed. The aim of this study was to assess the phytotoxicity of steel slags (SS) and concrete mixtures cast with a partial replacement of SS (CSS). Leaching tests were carried out on four SS and four CSS according to EN 12457-2 and UNI EN 15863, respectively. Each leachate was assayed using root elongation tests on 30 seeds of *Allium cepa*, *Cucumis sativus*, and *Lepidium sativum*, respectively, and on 12 bulbs of *A. cepa*. The latter also allowed the analysis of other macroscopic parameters of toxicity (turgidity, consistency, colour change and root tip shape) and the evaluation of the mitotic index on 20,000 root tip cells per sample. None of the samples induced phytotoxic effects on the organisms tested: all samples supported seedlings emergence, verified by root elongation comparable to, or even greater than, that of the negative controls, and did not affect cell division, as evidenced by mitotic index values. The absence of phytotoxicity demonstrated by the leachates allows SS and SS-derived concrete to be considered as reliable materials suitable for use in civil constructions or in other engineering applications, with economic and environmental advantages, such as the reduction of the final disposal in landfills as well as the consumption of natural resources.

The quality and type of steel produced by steel mills is regulated through the adjustment of incoming scrap metal. As a consequence, the derived steel slags (SS) have distinct chemical, mineralogical and physical characteristics (Cornacchia et al. 2015). In the sole Europe in 2018 the production of SS amounted to 16.3 million tons (Harder 2020). Due to their composition, mainly characterized by oxides of calcium, iron, silicon, aluminum, magnesium, and manganese (Cornacchia et al. 2015; Yüksel 2017), SS can be assimilated to natural hard rocks. SS could be reused in several applications such as soil amendment (Radić et al. 2013; Das et al. 2020; Kong et al. 2023), road base, asphalt mixtures (Paul et al. 2021), and in concrete production (Rondi et al. 2016; Collivignarelli et al. 2020). This last application is promising, due to the technical characteristics, good workability and mechanical properties of SS (Diotti et al. 2021). The reuse of such material, ultimately reduces its final disposal in landfill, thus limiting the consumption of natural resources. Nevertheless, the use of SS or SS-based materials may generate concern about the potential release of detrimental compounds into the environment. As a matter of fact, the characteristics of the steel production process could enrich the SS with potentially toxic elements, such as chromium, molybdenum, and vanadium (Primavera et al. 2016; Gan et al. 2022). To address this issue, which has not yet been plainly defined from the regulatory point of view, previous studies were developed on the toxicity and genotoxicity of granular SS leachates on living organism models representing different trophic levels (Alias et al. 2021). Among all the available model organisms, plants in particular are worth considering. Plant-based assays have a number of advantages, including high sensitivity, good correlation with animal systems, ecological pertinence, simplicity of execution, and, last but not least, low cost. Since decades, higher plants have been considered valuable systems for screening and monitoring environmental pollutants (Grant 1994). The United Nations Environment Program (UNEP), together with the World Health Organization (WHO) and the US Environmental Protection Agency (USEPA), promoted these tests by means of the International Program on Plant Bioassays (IPPB) (Roccotiello et al. 2011). Not less important, the diffusion of plant-based assays could improve public understanding of the toxic and genotoxic effects of pollutants (Ma 1999). These assays make it possible to thoroughly analyze the environmental degradation caused by a great variety of pollutants: air pollution (Hasanovic et al. 2022), pesticides (Menzyanova et al. 2022), plastic particles (Bouaicha et al. 2022), rare earth elements (Egler et al. 2022), urban and industrial wastewater (Bertanza et al. 2021; Chowdhary et al. 2022), municipal solid waste leachates (Palm et al. 2022), bottom ash and slag from municipal solid waste incinerators (Phoungthong et al. 2016; Tintner et al. 2016). Furthermore, plants make it possible to perform analyses on different tissues (leaves, roots, pollen), as well as to assess toxicity on the organism at different times in its life cycle. Among plant assays, those based on the measurement of seed root elongation provide an informative and rapid assessment of the environmental hazard of chemicals (substances or mixtures). This type of test can be useful for studying the chronic toxic effects of persistent substances in aqueous matrices. The USEPA Guidelines recommend using seeds of plant species of economic or ecological importance (USEPA 1996). In compliance with the ecotoxicology principle of “battery”, the regulatory institutions indicate from two or three (APAT 2004; ISO 18763,2016) up to ten or more (USEPA 1996) organisms belonging to both monocot and dicot classes. Among others, the common onion (*Allium cepa*), and the cucumber (*Cucumis sativus*) are frequently recommended by international and national agencies (USEPA 1996; APAT 2004; OECD 2006). Another well-recognized model in ecotoxicological assessments is represented by the garden cress (*Lepidium sativum*), commonly used to evaluate soil pollutants, sludge, and industrial waste (Methneni et al. 2021; Nikolaeva et al. 2021; Bożym 2022; Bona et al. 2023). The aim of this study was to assess the phytotoxicity of granular SS and SS-containing concrete leachates by using a battery of plant-based tests composed of seeds of *A. cepa*, *C. sativus*, *L. sativum*, and bulbs of *A. cepa*.

## Materials and Methods

Four samples of steel electric arc furnace (EAF) slags (SS1, SS2, SS3, SS4) were collected from four different steel mills in northern Italy. Each plant had an adequate input of scrap metal to obtain structural steel (plant 1) or special steel (plants 2, 3 and 4), which implies the production of different steel slags. Steel slags were stored by the factories in open, unprotected areas for at least 90 days.

Four concrete mixtures, called concrete-SS (CSS), were cast with the use of Portland cement (13% w/w) and a partial replacement (30% w/w) of natural aggregate with SS. The cement/water ratio was 0.48 and the concrete density was about 2470 kg/m^3^. Casts were naturally dried to constant weight (approximately 30 days) prior to analysis.

SS was submitted to EN 12457-2 leaching test (EN 12457-2 2002) performed by mixing the homogenized sample with demineralized water at a liquid to solid ratio of 10 L/kg. The mixture was placed on a tightly closed rotary shaker (VELP, Italy) and agitated for 24 h, rotating at 10 ± 2/min. The solutions were filtered (0.45 μm) and stored at 4 °C. CSS leaching test was performed following the standard procedure on building products (UNI CEN/TS 16637 2014), for the first two extraction stages only (total duration 24 h) which can reasonably be considered to represent the most critical situation for the release of pollutants. Tests were performed on soaked concrete blocks in demineralized water (leachant) at a liquid to surface area ratio of 8 mL/cm^2^. The leachant was renewed after 6 and 18 h of contact. At the end of these periods, the solutions were filtered (0.45 μm), the pH was measured using a pH glass electrode (Hanna Instruments, Italy) according to ISO 10523, and the electrical conductivity (EC) was determined by a platinum potentiometric probe (Hanna Instruments, Italy) according to ISO 7888. The leachates were then stored at 4 °C.

The root elongation assays on seeds were performed following the Italian standard with some modifications (UNI 11357,2010). Briefly, seeds of onion (*A. cepa*), cucumber (*C. sativus*), and garden cress (*L. sativum*), not treated with fungicides, were preliminarily checked for vitality in distilled water in the dark at 25 ± 1 °C (germination rates > 90%). Leachate solutions were tested without any dilution, and the distilled water was used as negative control. Three replicates per treatment were arranged by wetting a Whatman no. 1 filter paper with 2 mL of each solution. Ten seeds for each replicate were distributed on the filter. The three dishes of each replicate were packed into a tightly closed plastic bag and incubated at 25 ± 1 °C in the dark for 72 h. At the end of the incubation time, the root length of the sprouts (≥ 1 mm) was assessed.

The root elongation test on bulbs was performed on equal-sized young onion bulbs purchased from the local market without any treatment. Twelve bulbs were exposed for 72 h in the dark to leachate solutions without any dilution, and the distilled water was used as negative control. The roots’ mean length was calculated (Fiskesjö 1995). Results were expressed as mean length ± SD. Other toxicity parameters (turgidity, consistency, colour change and root tip shape) were also evaluated. Moreover, root tips were cut and stained with 2% acetic orcein to assess the mitotic index (MI), a proxy for the cell division ratio. The microscopic analysis (1000 × magnification) was conducted on 20,000 cells per sample.

The experiments were performed in duplicate. The statistical analysis was performed using Student’s t test, where *p* < 0.05 was considered significant.

## Results

The chemical parameters pH and EC of leaching solutions are summarized in Table 1. The SS leachates demonstrated the expected alkalinity with pH ranging from 8.5 to 10. The leachates from CSS displayed lower pH values than the corresponding granular SS, though in the alkali range (8.0–9.0). The pH value of sample CSS1-6h was the only exception in that it was neutral. The EC of SS showed some variability, ranging from 119.3 µS/cm to 342.0 µS/cm of samples SS2 and SS1, respectively, while samples SS3 and SS4 were both slightly above 200 µS/cm. The EC values of leachates from CSS showed higher consistency among the two fractions of leachates. Moreover, a slight increase over time was observed, with the lower value shown by CSS2-6h (78.0 µS/cm) and the higher EC values obtained by CSS3-18h (126.0 µS/cm).

**Table 1:** Chemical parameters pH and EC of leaching solutions from SS and CSS

Sample	SS		CSS			
			6 h		18 h	
	pH	EC (µS/cm)	pH	EC (µS/cm)	pH	EC (µS/cm)
1	9.0	342.0	7.0	102.7	8.0	117.7
2	10.0	119.3	8.0	78.0	8.5	107.1
3	8.5	220.0	8.0	109.0	8.0	126.0
4	9.5	208.3	9.0	123.4	8.5	125.6

**Fig. 1:**
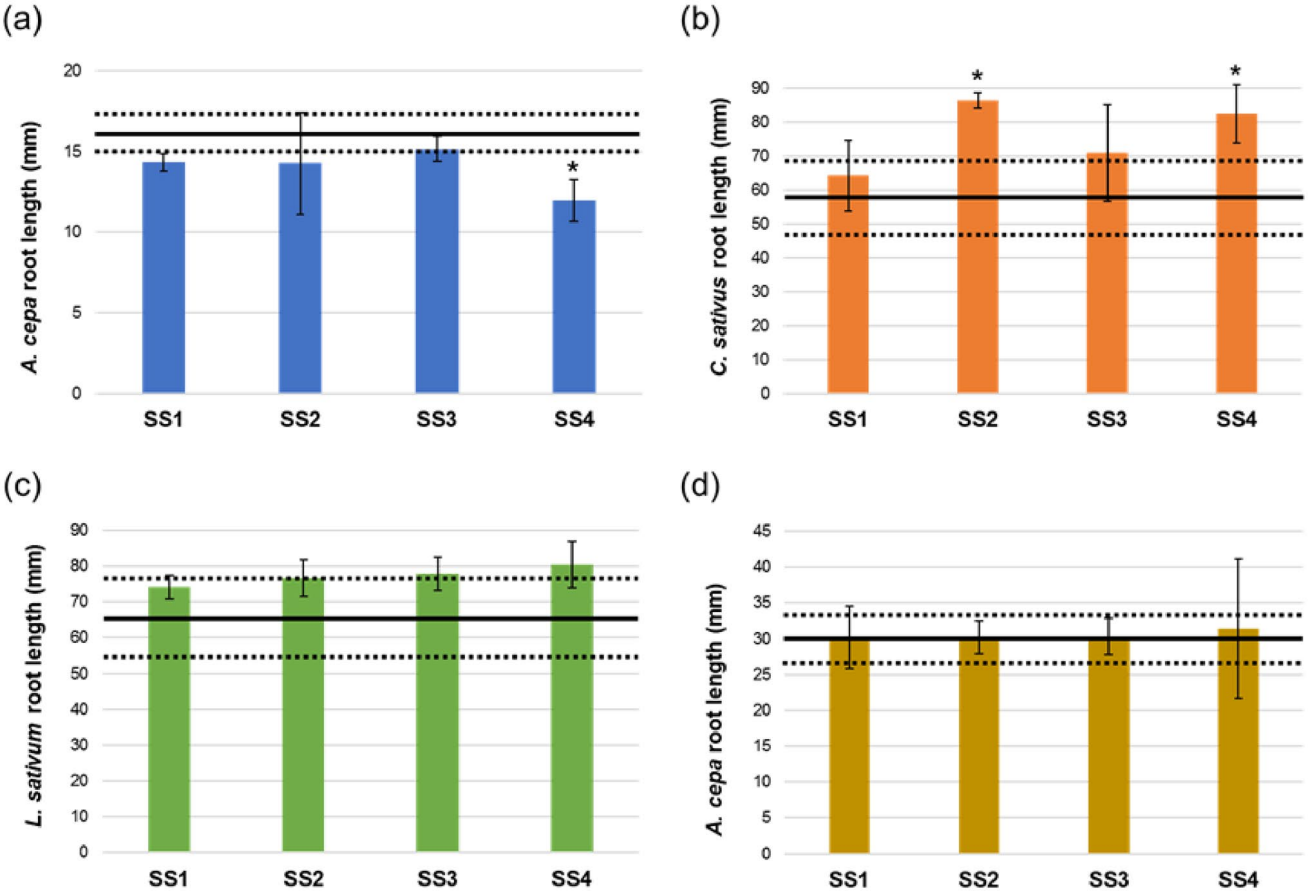
Root elongation of **a** *A. cepa*, **b** *C. sativus*, **c** *L. sativum* seeds, and **d** *A. cepa* bulbs treated with undiluted leachates of SS obtained according to UNI 12457-2. Data are expressed as mean ± SD. Bold line represents mean root length of negative control ± SD (dotted lines). *Statistically significant versus negative controls according to Student’s *t* test (*p* < 0.05)

The phytotoxicity of leachates obtained from SS was assessed through the root elongation of *A. cepa*, *C. sativus*, and *L. sativum* seeds and *A. cepa* bulbs (Fig. 1). None of the samples caused significant toxic effects on both seeds and bulbs. The only exception was a slightly significant inhibition effect induced by sample SS4 (12.0 ± 1.3 mm) in *A. cepa* seeds (Fig. 1a). Conversely, samples SS2 and SS4 caused a significant stimulation of root elongation in *C. sativus* (86.3 ± 2.4 and 82.4 ± 8.5 mm, respectively) (Fig. 1b).

Table 2 summarizes the macroscopic parameters and mitotic indexes assessed on the roots of onion bulbs exposed to SS leachates. The macroscopic parameters (turgidity, consistency, color change and root tip shape) of onion bulbs roots were mostly normal, with the exception of the presence of the “hook” root shape. In particular, sample SS3 induced the highest frequency of hooks (33%), while samples SS1, and SS4 were perfectly comparable to the negative control (17%). Surprisingly, sample SS2 did not induce any root modification. Furthermore, the assessment of the MI of the onion bulbs roots confirms the absence of any effect on cell division, as no differences were found between the MI of the roots exposed to SS samples (from 10.02% to 11.46%) and that of the negative control (12.65%).

**Table 2:** Macroscopic parameters (MP) and mitotic indexes (MI) of *A. cepa* bulbs roots treated with undiluted leachates of SS

Samples	MP (%)	MI (%)
	Hooks	
SS1	17	11.41
SS2	0	11.46
SS3	33	10.39
SS4	17	10.02
Neg. control	17	12.65

**Fig. 2:**
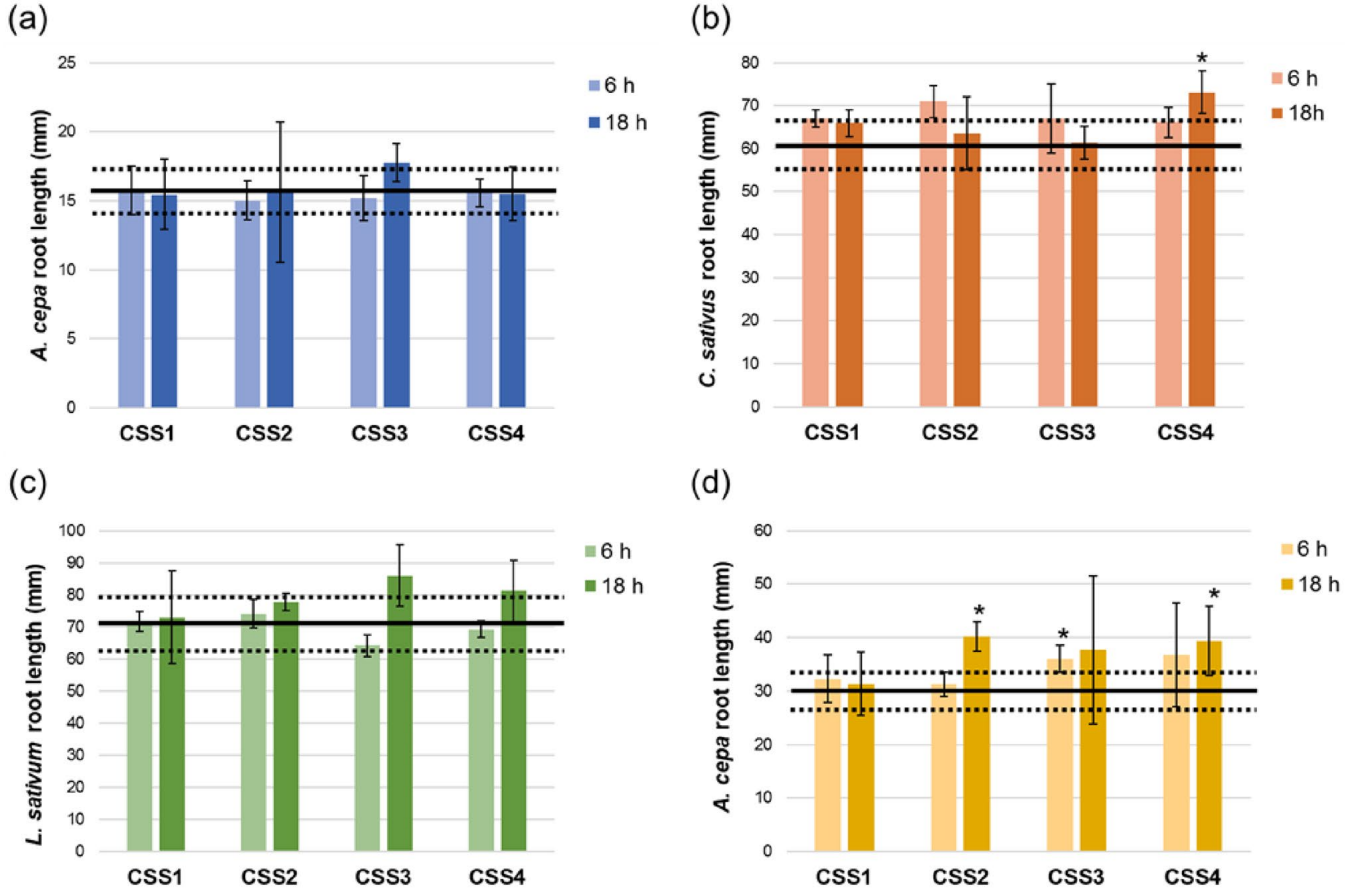
Root elongation of **a** *A. cepa*, **b** *C. sativus*, **c** *L. sativum* seeds, and **d** *A. cepa* bulbs treated with undiluted leachates of CSS obtained according to UNI EN 15863. Data are expressed as mean ± SD. Bold line represents mean root length of negative control ± SD (dotted lines). *Statistically significant versus negative controls according to Student’s *t* test (*p* < 0.05)

The phytotoxicity of leachates obtained from concrete cast with a partial replacement of SS was than assessed (Fig. 2). Similar to the previously described samples, the leaching fractions obtained after 6 and 18 h of contact did not exert toxic effects on plant species, as demonstrated by the root elongation which was not significantly reduced compared to the negative controls. On the other hand, some samples significantly increased root elongation: sample CSS4-18h acted positively on both *C. sativus* (73.1 ± 4.9 mm) and *A. cepa* bulbs (39.4 ± 6.5 mm). The latter was also stimulated by samples CSS2-18h (39.8 ± 2.9 mm), and CSS3-6h (36.0 ± 2.5 mm). Table 3 summarizes the macroscopic parameters and mitotic indexes assessed on onion bulbs roots exposed to CSS leachates. Again, the macroscopic parameters of onion bulbs roots were mostly normal, with the exception of the presence of two root shapes: “hooks” and “broken”. In particular, the former was induced by all samples (from 17% to 33%), except for CSS4. The “broken tips” were only generated by the 18 h fraction of samples CSS1 and CSS3. As shown for the previous data on SS, the assessment of the MI confirmed the absence of any effect on cell division, as no differences were found between the MI of the roots exposed to the samples (from 10.06% to 11.34%) and that of the negative control (10.90%).

**Table 3:** Macroscopic parameters (MP) and mitotic indexes (MI) of *A. cepa* bulbs roots treated with undiluted leachates of CSS

Samples	MP (%)		MI (%)
	Hooks	Broken tips	
CSS1-6 h	17	0	10.66
CSS1-18 h	17	33	11.10
CSS2-6 h	20	0	10.71
CSS2-18 h	33	0	10.79
CSS3-6 h	33	0	11.34
CSS3-18 h	17	17	10.58
CSS4-6 h	0	0	10.06
CSS4-18 h	0	0	11.00
Neg. control	20	30	10.90

## Discussion

Leachates from SS and from the concrete cast with a partial substitution of SS were characterized through a plant-based approach to assess their impact on terrestrial compartment. Although the lack of detailed chemical data on leachates may be a limitation of this research, this study mainly focused on the effects induced by leachates, which are a complex mixture enriched by metals and ions species, on plants. Previous studies conducted on other batches of slags from the same steel mills, revealed that the leachate solutions had a low chemical content, below the limit values imposed by the Italian legislation for the recovery of non-hazardous waste (Alias et al. 2021). Interestingly, both SS and CSS leachates showed an overall comparable non-toxic behaviour on plants. This suggests that the presence of industrial waste in concrete does not worsen the impact and may even lower the toxicity of the waste alone on plant organisms. The immobilization of SS in concrete produced a clear reduction in toxicity, as observed between SS4 and CSS4 sample treatments in *A. cepa* seeds. Recently, Brás et al. showed that concrete incorporating industrial waste is safer than reference concrete, by measuring the toxicity in terms of duckweed (*Lemna gibba*) fronds growth (Brás et al. 2020). Moreover, the effects of the two subsequent fractions of leachates (after 6 and 18 h of contact) suggest the release of a non-toxic mixture within the first 24 h of analysis. As surface wash-off and dissolution were identified as the predominant leaching mechanisms for several pollutants (e.g. heavy metals) in monolithic samples (Kogbara et al. 2013), more leaching fractions (up to 64 days) should be evaluated to better investigate the potential release or accumulation over time of substances with toxic or biostimulation properties. This latter suggestion, also made by Brás et al. 2020, is relevant as leachates from concrete and slag are known to be rich in minerals required for plant growth (e.g. calcium, silica, magnesium). In the same vein, Ishimori et al. have lately investigated the feasibility of using waste concrete leachates instead of fertilizers (Ishimori et al. 2022). Plants represent valuable toxicological models for environmental studies due to their strong ability to interact directly with pollutants and to tolerate a wide range of experimental conditions, especially in terms of pH, salinity and temperature (Grant 1994). The phytotoxicity assays on *A. cepa, C. sativus,* and *L. sativum* seeds and *A. cepa* bulbs were performed without any modification of the leaching solutions (dilutions or pH adjustment) and the observed effects appear to be independent from their pH and EC. Given that the evaluation of an unmodified sample is a possibility allowed by few toxicological assays, this observation makes plants pivotal organisms in the ecotoxicology of construction materials, where the analysis of leachates without any adjustment is crucial for a more realistic assessment of their environmental impact (Mocová et al. 2019). Interestingly, plant-based tests also represent a powerful tool for toxicology in line with the application of the 3Rs principles (Russell and Burch 1959), because of their high concordance with animal systems, including humans (Tedesco and Laughinghouse IV 2012; Reis et al. 2017). This means that plant assays could provide a valuable assessment of the toxicity and even genotoxicity of different samples (particularly in the environmental field), avoiding or limiting the use of animals. Recently, the international bioscience community has been gone ahead, adopting the expression of new approach methodologies (NAMs) to highlight the importance of non-animal technologies (NATs) use in chemicals, drugs, food, and environmental toxicity assessment (Van Mulders et al. 2022). Tests on seed models have additional multiple advantages, such as ease of execution, low cost, wide range of available species, and, above all, sensitivity to heavy metals and organic pollutants, as observed by Bozym et al. in their study of foundry dust and landfilled waste on *L. sativum* seeds (Bożym 2022). On the other hand, seeds are also able to discriminate non harmful matrices, as demonstrated by Bona et al. who investigated hydrochar from digestate and hydrochar co-compost through an analysis of root modifications on *L. sativum*, *C. sativus*, and *Sorghum bicolor* (Bona et al. 2022). In this study, *A. cepa* (monocot) and *L. sativum* and *C. sativus* (dicots) were used because of their variable sensitivity to potentially heavy metal-polluted samples and their representativeness of agronomically relevant fruit and vegetables species. As already observed by Baderna et al. dicots and a monocot responded differently to metals and metal mixtures (Baderna et al. 2015). All of this reinforces the idea that the obtained results on SS and CSS were not due to a lack of sensitivity of the test models, but to a proper non-phytotoxicity of the samples. The effects of the tested samples on the macrophytes used showed only slight differences, both between the different classes (monocot *A. cepa* versus dicots *C. sativus* and *L. sativum*) and between different forms of the same plant (seeds versus bulbs of *A. cepa*). This demonstrated how consistency between the results of a battery of plant assays supports the data obtained and allows for the identification with higher certainty of samples of greater or lesser concern.

Furthermore, plant-based tests, as reliable and sensitive systems, could overcome the limits of the chemical analyses which detect single and known elements, revealing the synergistic effects of mixtures characterized by low or very low concentrations of several compounds. More generally, biological assays are powerful tools because of this independence from a pre-determined analytical schedule, which does not necessarily describe a sample completely (Kortenkamp and Faust 2018; Escher et al. 2020; Luo et al. 2022). Put simply, a synthesis could be the motto: “It does not matter what the chemical composition is, but rather its effect on living organisms”. Furthermore, ecotoxic assessments could help to correctly manage a waste product, shifting the reusing from a system with a hazardous impact (e.g., some granular waste) to a less harmful one (e.g., the same waste product incorporated into inert matrices), thus minimising disposal and landfilling to highly hazardous waste only. The transition from waste to product feeds the virtuous path of the circular economy. This could be an interesting prospect for the steel industry, which would have direct economic advantages. Not least, this could generate environmental benefits due to the lower demand for primary resources.

## Conclusion

The findings presented here did not reveal any concerning aspect of the tested samples, thus supporting the idea that not only the use of granular SS but also their application in the construction field could play a role in making industrial activities more sustainable towards the environment. This study contributes to the ongoing debate on waste management and the decision-making process regarding the reuse of such materials, shifting the perspective from a “static” chemical characterization of a waste to a toxicological one, that describes the effects on living organisms as a result of dynamic processes.
